# Marine Sediments Remotely Unveil Long-Term Climatic Variability Over Northern Italy

**DOI:** 10.1038/srep12111

**Published:** 2015-07-31

**Authors:** Carla Taricco, Silvia Alessio, Sara Rubinetti, Davide Zanchettin, Simone Cosoli, Miroslav Gačić, Salvatore Mancuso, Angelo Rubino

**Affiliations:** 1 Dipartimento di Fisica, Università di Torino, Italy; 2 Osservatorio Astrofisico di Torino, INAF, Pino Torinese, Italy; 3 Università Ca’ Foscari, Venezia, Italy; 4 Istituto Nazionale di Oceanografia e Geofisica Sperimentale (OGS), Sgonico, Trieste, Italy

## Abstract

A deep understanding of natural decadal variability is pivotal to discuss recently observed climate trends. Paleoclimate proxies allow reconstructing natural variations before the instrumental period. Typically, regional-scale reconstructions depend on factors like dating, multi-proxy weighting and calibration, which may lead to non-robust reconstructions. Riverine records inherently integrate information about regional climate variability, partly overcoming the above mentioned limitation. The Po River provides major freshwater input to Eastern Mediterranean, as its catchment encompasses a large part of Northern Italy. Here, using historical discharge data and oceanographic measurements, we show that Po River discharge undergo robust decadal fluctuations that reach the Ionian Sea, ~1,000 km South of Po River delta, through propagating salinity anomalies. Based on this propagation, we use a high-resolution foraminiferal δ^18^O record from a sediment core in the Ionian Sea to reconstruct North Italian hydrological variability on millennial-scale for the first time. The reconstruction reveals highly significant decadal variability that persists over the last 2,000 years. Many reconstructed extremes correspond to documented catastrophic events. Our study provides the first millennial-scale reconstruction of the strength of decadal hydrological variability over Northern Italy. It paves the way to assess the persistence of large-scale circulation fingerprints on the North Italian climate.

Climate variability in the Mediterranean region is deeply influenced by meridional displacements of the trajectories of large perturbations originating over the Atlantic^1^,^2^.

The Adriatic Sea is particularly sensitive to this mode of variability due to its peculiar morphology and location within the Eastern Mediterranean (Fig. 1a). Strong oceanic phenomena occur in the Adriatic like, e.g., persistent coastal currents as well as shelf and open-ocean deep convection. These are largely induced by the prominent forcing of the opposite winds Sirocco and Bora, and the conspicuous and remarkably time-varying inflow of Alpine riverine freshwaters dominated by the Po River^3^, which alone contributes to about half of the total freshwater inflow into the Adriatic^4^.

Po River discharges and North Italian precipitation were characterized, during the last century, by large quasi-synchronous fluctuations^5^ (Fig. 2a,b). The persistence of this coherence over the last 140 years indicates that precipitation variability dominates on direct human interventions in the basin, strong anthropization of the Po River catchment and associated alteration of discharge processes through, e.g., land use alterations^6^.

A spectral analysis of the Po discharge series reveals that a decadal component constitutes its dominant mode (see Supplementary information). Indeed, decadal-scale variability accounts for about 13% of total variability of annual-average Po River discharges during the 20^th^ century. Within these decadal fluctuations, the Po River provides on average about 1,730 m^3^/s of freshwater into the Northern Adriatic during wet periods, while average discharges only slightly exceed 1,300 m^3^/s (Fig. 2a) during dry periods. Additional contributions from other major co-varying Alpine rivers amplify this difference (see Supplementary information).

In the shallow Northern part of the Adriatic Sea, hydrodynamics and oceanographic features highly depend on seasonal air-sea heat fluxes, wind forcing and local freshwater inflow^7^, which is dominated by the buoyant plume of the Po. Its formation, shape and evolution are strongly influenced by seawater stratification and prevailing winds, and deeply influence the larger-scale circulation in the basin. In particular, the Po plume can be mostly confined to the Western Adriatic flank, especially under typical winter conditions (November to March), or it can be entrained into the wind-induced Northern cyclonic gyre as a consequence of prolonged intense Bora events^8^. Sirocco can reverse the Western coastal current and advect Po waters towards the Northern coast and offshore^9^. However, the fate of the signal of this large haline and, during winter, thermal anomaly is its substantial weakening while transported along the Italian shelf toward the Southern entrance of the basin and, then, the Ionian Sea.

There is strong evidence for the existence of decadal fluctuations affecting this circulation pattern^10^ both at the surface and in intermediate layers, where an inflow of Levantine Intermediate Water takes place along the basin’s Eastern flank. In the near-bottom layers, dense waters generated in the Northern part of the basin (and hence influenced by the local freshwater input as well) outflow along the Western flank toward the Ionian Sea^11^. Hence, temperature and salinity anomalies induced in the Northern Adriatic are advected southwards along the western Adriatic coast in the form of a swift coastal flow.

This phenomenon, if demonstrated quantitatively, would provide the physical basis to reconstruct North Italian hydro-climatic variability over the last two millennia using a high-resolution record of foraminiferal δ^18^O from a sediment core of the Ionian Sea.

## Results

**Figure 1:**
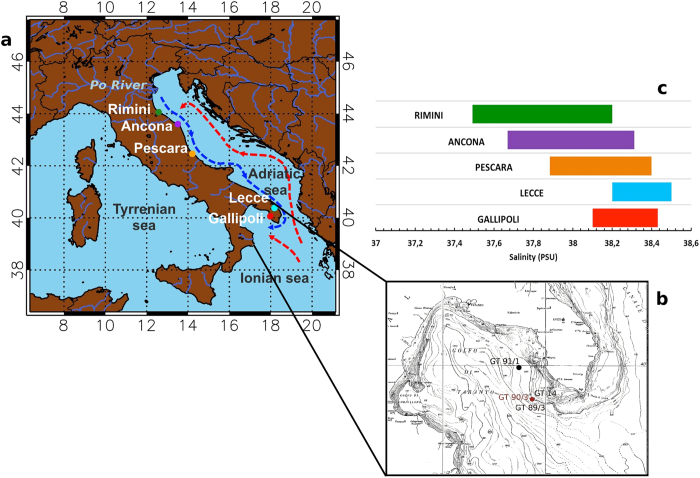
Amplitude of salinity decadal variability along the Adriatic coast. (**a**) Map of the Adriatic Sea with surrounding regions (made with the Interactive Data Language (IDL) software, IDL Version 8.3 EULA, Exelis Visual Information Solutions, Inc., Boulder, Colorado, USA), showing the location of the Po River and the path of the freshwater outflow along the Italian coastline, the dominant path of the inflowing currents along the Croatian coasts, and a schematic circulation pattern in the Gulf of Taranto. (**b**) Bathymetric map showing the Gallipoli Terrace in the Gulf of Taranto, Ionian Sea, and the location of the sediment cores studied by our group (see points on the map). The isotopic ratio δ^18^O was measured in core GT90/3 (red point on the map), drilled at (39° 45’ 53” N, 17° 53’ 33” E); the core was extracted at a depth of 178 m and has a length of 3.57 m. (**c**) Amplitudes of decadal variability of upper-layer salinity measured since the early 1940s in selected locations along the Eastern Italian coast (ranked from North to South).

**Figure 2:**
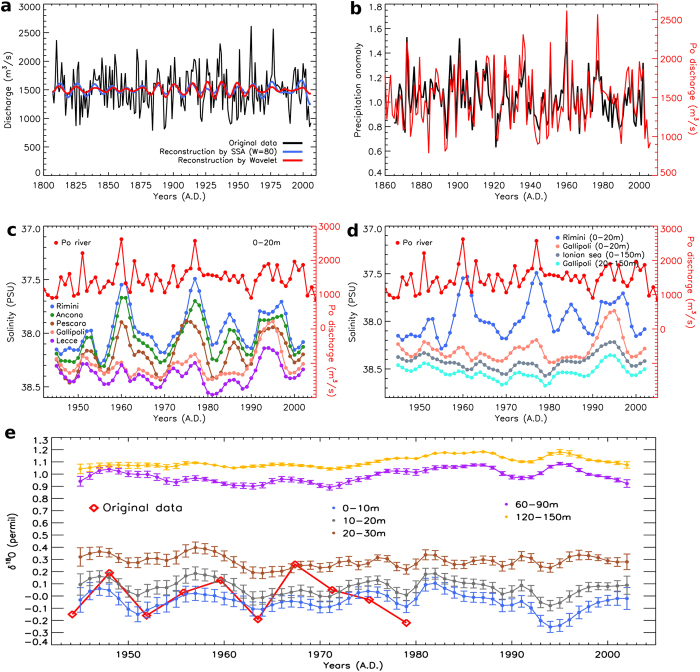
Decadal fluctuations in Po River discharges, salinity profiles from the Western Adriatic and Northern Ionian seas, and δ^18^O series from the GT90/3 Ionian Sea core. Panel **a**: Annual-average Po River discharges and associated decadal oscillations estimated by SSA (blue curve) and CWT (red curve), as described in the Supplementary information. Panel **b**: Average precipitation^32^ over the Po valley (relative deviations). Panel **c**: Upper-layer (0–20 m depth) salinity measured along the Western Adriatic coast. Panel **d**: Salinity at Gallipoli (at 0–20 and 0–150 m depths), Rimini (0–20 m depth) and in the Northern Ionian Sea (South of Calabria, 37° 40’ N, 16° 20’E, 0–150 m depth). Panel **e:** δ^18^O values (red points) measured in G. ruber of the core GT90/3 extracted from the Gallipoli terrace (Ionian sea) and δ^18^O of the calcite calculated using the Shackleton equation with salinity and temperature values at different depths corresponding to the same Gallipoli site (plotted anomalies are with respect to the average value of the 0–20 m layer).

Historical salinity data allow to demonstrate the phenomenon described above^12^. Strong salinity anomalies reveal prominent decadal variability along the whole Western Adriatic coast and in the Northern Ionian Sea: generally amplitudes weaken southwards (Fig. 2c, see also Fig. 1c). The decadal fluctuations are quasi-synchronous with those observed in the Po River discharge data (Fig. 2c), which support the Northern riverine origin of the decadal salinity signal observed along the Adriatic coast and in the Gulf of Taranto. A slight increase in the amplitude of fluctuations from the Adriatic (Lecce) to the Ionian (Gallipoli) also occurs. Salinity fluctuations in the Gulf of Taranto are not always in-phase with those in the Adriatic Sea, suggesting that other factors also contribute to the observed local variability. Analysis on freshwater surface fluxes from reanalysis data excludes local evaporation and precipitation as a major factor for the observed salinity fluctuations (see Supplementary information).

Instead, they can be explained by the Adriatic—Ionian feedback mechanism named BiOS (Bimodal Oscillating System)^13^, which consists of the alternating inflow into the Northern Ionian and the Adriatic of relatively saltier waters of Levantine origin and relatively fresher waters from the Atlantic Ocean. The influence of BiOS on the observed data is supported by the presence, in the 1990s, of the most prominent salinity minimum of the entire records in the Ionian Sea (grey curve in Fig. 2d). Such minimum is not prominent in the Adriatic Sea (Fig. 2c), except in its southernmost portion (Lecce; violet curve in Fig. 2c). This event can be associated with a strong anticyclonic circulation of the North Ionian Gyre and the inflow of the low-salinity waters of Atlantic origin.

A high-resolution and accurately dated (see Methods) record of foraminiferal δ^18^O isotopic ratio measured in the sediment core GT90/3 extracted from the Gallipoli Terrace in the Gulf of Taranto (Ionian Sea, see Fig. 1b) reveals a highly significant decadal oscillation, persistent over the last 2,000 years^14^.

Planktonic foraminifera are strongly influenced by the environmental conditions of the near-surface ocean in which they live, particularly due to their sensitivity to ambient temperature and salinity. Different temperature-salinity conditions generate different isotopic values of δ^18^O in the foraminifera shells^15^,^16^. The Gallipoli δ^18^O record agrees well with a δ^18^O theoretical estimation based on observed near-surface temperature and salinity in the vicinity of the core location (see Fig. 2e and Methods).

The theoretical δ^18^O series of Fig. 2e show that: a) decadal variations are present in the upper layers; b) notwithstanding the different temporal resolution, these variations are in phase with the foraminiferal δ^18^O (red points) and the experimental amplitudes (0.2–0.3 permil) are in good agreement with the theoretical estimates derived for the layer in which these organisms dwell, i.e. 0 to 20 m depth; c) the amplitude of δ^18^O variability decreases with depth and the decadal oscillation is completely damped below about 30 m depth, thus confirming that it arises from the near-surface layers.

As we mentioned before, the analysis by different spectral methods reveals a highly significant decadal cycle with modulated amplitude, throughout the whole 2,200-yr long δ^18^O record (see Supplementary information). The persistency of these decadal oscillations over 2 millennia suggests that the underlying mechanisms are active over such a long period and that the Alpine riverine freshwater inflow, largely determined by Po River discharge variations, is likely a leading one, since the decadal component dominates the total variability of the Po discharge series (Fig. 2a). Moreover, the Po River origin of the decadal oscillation is also supported by the northward increase of the decadal salinity signal.

**Figure 3:**
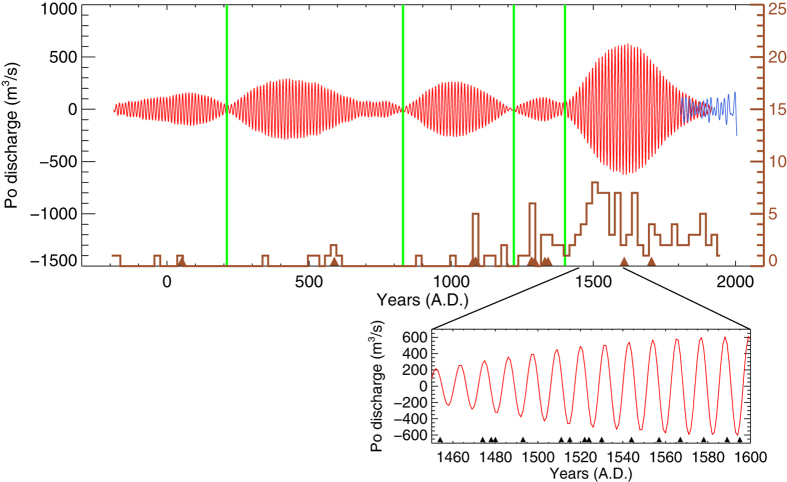
Extrapolation of the Po River discharge before 1917 to the last 2 millennia (red curve). As a reference for the last centuries, the Po discharge decadal component is superimposed (blue curve). Known major floods in the Po plain from 200 BC^17^ are represented by the histogram and the major ones by black triangles. The same weight is attributed to each flooding event, since documental description is only qualitative. An expanded view of the reconstruction for the 16^th^ century (bottom panel) reveals an outstanding agreement between the exceptional events documented during this period (black triangles)^20^ and the reconstructed phases of maximum discharge.

Based on the relationship between δ^18^O and Po River discharge decadal variability observed over the last 70 years (see Methods), we reconstructed the latter over the 2,200 years covered by the δ^18^O record (see Fig. 3). The amplitude of the reconstructed Po River discharge decadal fluctuations only sporadically falls below 50 m^3^/s (Fig. 3), suggesting that decadal variability is a robust and substantial component of Po River discharge total variations. During the last two millennia, we notice large amplitudes over prolonged periods, especially between 300 and 600 AD, around 1000 AD and between 1400 and 1800 AD. The highest hydrological variability of the last two millennia is detected around 1600 AD, in coincidence with the coldest phase of the Little Ice Age (LIA). The large discharge excursions reconstructed during this period have amplitudes comparable with those observed in the last century (see events around 1936, 1960, 1977 in Fig. 2a). This suggests that a long (centennial-scale) sequence of high discharge events during the LIA might have caused the reconstructed maximum of the amplitude modulation around 1600 AD.

## Discussion

The Po discharge reconstruction showed in Fig. 3 relies on three assumptions: linearity of the extrapolation, stationarity of the relation over 2,200 years and adequacy of the calibration period.

The assumption of stationarity of the δ^18^O-Po River discharge relationship implicitly contains the assumption of stationarity of the relationship between δ^18^O and salinity of the waters in which the foraminifera live. So, we consider the freshwater signal propagation along the Western Adriatic coast into the Ionian Sea as a persistent and dominant feature of oceanic variability in the Eastern Mediterranean.

Furthermore, the Po River discharge signal is certainly far better represented by salinity variations whereas δ^18^O reflects both temperature and salinity conditions, with comparable dependence. While the linkage between salinity and Po River discharge is straightforward, it is not so for temperature, since waters affected by the Po River do not have a distinct unambiguous temperature signature. Therefore departures from the dependence of δ^18^O on the Po River discharge rate can be attributed to the temperature influence or also to a salinity variation related to occasional reversals of the Ionian circulation.

Regarding the assumption of adequacy of the calibration period, it is again related to our limited knowledge about centennial and longer Po River discharge variability and its imprint on the Adriatic and Ionian Sea circulation. Moreover, we have limited knowledge of centennial and longer oceanic variability in the Mediterranean Sea: for instance, actually the effects of the BiOS mechanism are known only for the period since 1940s.

Moreover, although the Po River discharge record covering the last two centuries shows weak spectral power at low frequencies, we cannot exclude the presence of substantial centennial-scale variability during the last two millennia. With such caveats, this reconstruction represents a unique opportunity to get information about the Po variability, hence on aspects of climate in a large part of the North Italian Peninsula over such a long time interval.

Archival reports^17^ on floods in the Po basin during the last two millennia corroborate the hydrological variations depicted by our reconstruction. The major documented floods (brown triangles in Fig. 3) occur during periods characterized by strong decadal variability in our reconstruction. For instance, the maximum reconstructed amplitudes occur around the 16^th^ and 17^th^ centuries and correspond to three documented extreme events in 1609, 1704 and 1705 AD. The histogram in Fig. 3 (brown line) summarizes the occurrences of all events documented since 200 BC (we attributed the same weight to each event, since documental description is only qualitative). Periods of weak reconstructed amplitude are found in our reconstruction around 200, 800, 1200 and 1400 AD and coincide with periods when no flooding event was documented. This result relies on the accuracy of the above mentioned archival reports^17^ and a possible lack of documented events -especially in the oldest portion of the series—has to be taken into account. However, the decrease of the number of documented events after 1700 AD is certainly not an artifact related to scarce information and strongly supports the reliability of the histogram maximum during the LIA. In addition, the record of flood frequency at Lake Ledro^18^,^19^ (Northern Italy, 45°N; see Fig. 3, panel c in Magny *et al.*, 2013) reveals that during the last two millennia the highest frequency was observed during the LIA, thus supporting the high variability shown by our reconstruction during this period.

Researches on climatic variations in the Po plain evidenced that the 16^th^ century was characterized by an exceptional series of floods which struck Northern Italy^20^. Moreover a high climate variability during that century results from the presence of very dry periods, often following years of heavy spring and autumn rains^20^. This provides evidence of generally disturbed hydrological conditions and of an increase in the occurrence of extreme events during the years 1530–1590 as compared with the preceding periods.

An expanded portion of Fig. 3 reveals an outstanding agreement between the exceptional events of that period (black triangles) and the maxima of our reconstruction. Combining information from both sources, alternating decadal wet and dry phases during this period led to a decadal recurrence of major flooding events.

So, the presence of strong decadal variability is supported not only by the relevance of this component in the Po discharge series, but also by the historical documentation of flood events during the last two millennia, especially during the 16^th^ century.

However, it is unclear whether our reconstruction truly reflects the alternation of wet and dry periods according to a decadal cycle, rather than a decadal occurrence of flooding events only. As we mentioned above, we cannot exclude the presence of substantial centennial or longer-scale variability in Po River discharges during the last two millennia, which would shift the mean discharge value with respect to the constant mean level of our reconstruction. This effect could, for example during the LIA, attenuate the discharge minima.

In conclusion, our study provides the first millennial-scale reconstruction of the strength of decadal hydrological variability over Northern Italy. It paves the way to assess the persistence of large-scale circulation fingerprints on the North Italian climate on millennial scales and hence to improve understanding of how variability modes influence regional climates in the Northern Hemisphere.

## Methods

### Tephroanalysis and radiometric core dating

The high accuracy of the dating of shallow-water Ionian Sea cores, drilled from the Gallipoli Terrace in the Gulf of Taranto (Fig. 1b), is made possible by the closeness of the drilling site to the volcanic Campanian area, a region that is unique in the world by its detailed historical documentation of volcanic eruptions over the last two millennia. The markers of these eruptions were identified along the cores as peaks of the number density of clinopyroxene crystals. The time-depth relation for the cores retrieved from the Gallipoli Terrace^14^,^21^,^22^,^23^,^24^,^25^,^26^ was obtained by tephroanalysis.

We found 22 sharp pyroxene peaks, corresponding to historical eruptions of the Campanian area, starting with the 79 AD eruption of the Vesuvius that buried Pompei and ending with its last eruption in 1944^27^.

**Figure 4:**
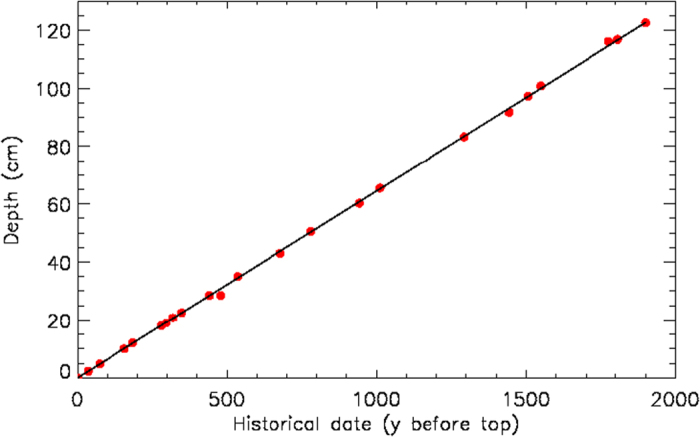
Tephroanalysis of sediments from the Gallipoli Terrace: time-depth relationship. The depth at which a volcanic peak is found in the sediment is plotted versus the historical date of the corresponding eruption, expressed in years counted backwards from 1979 AD, i.e. the date of the core top. The straight line resulting from a linear regression fit to the experimental data is also shown.

Figure 4 shows the time-depth relation over the last two millennia.

Each point represents a pyroxene peak found at a given depth, and corresponding to a historical eruption. The linear regression gives h = (0.0645 ± 0.0002)y_BT_ , where h is depth in cm, y_BT_ means year-before-top (top = 1979 AD) and the correlation coefficient is r = 0.99; the slope of this line is the sedimentation rate. This relatively high sedimentation rate allows high-resolution studies in time: the sampling interval of the core, 2.5 mm, corresponds to 3.87 y. The highly linear time-depth relation demonstrates that the sedimentation rate has remained constant, to a very good approximation, over the last two millennia. Moreover, the measurements performed in different cores retrieved from the same area showed that this rate is also uniform across the whole Gallipoli Terrace^22^,^23^,^25^. The very sharp pyroxene peaks indicate that bioturbation by bottom-dwelling organisms is quite limited; we thus conclude that the climatic record is not significantly affected by sediment mixing.

Taricco *et al.* (2008)^28^ confirmed this dating by applying advanced statistical procedures^29^,^30^.

The tephroanalysis dating confirmed, improved and extended to the deeper part of the core the dating obtained in the upper 20 cm by the ^210^Pb method^21^,^31^.

The ^210^Pb activity evaluation^21^ has been carried out at the low-level counting Laboratory of Monte dei Cappuccini (INAF) in Torino (70 m.w.e). The activity was determined though detection of the β^−^ activity of the ^210^Bi (T_1/2_ = 5.013 d) in equilibrium with ^210^Pb, using a set of Geiger-Müller operating in a shielded well.

**Figure 5:**
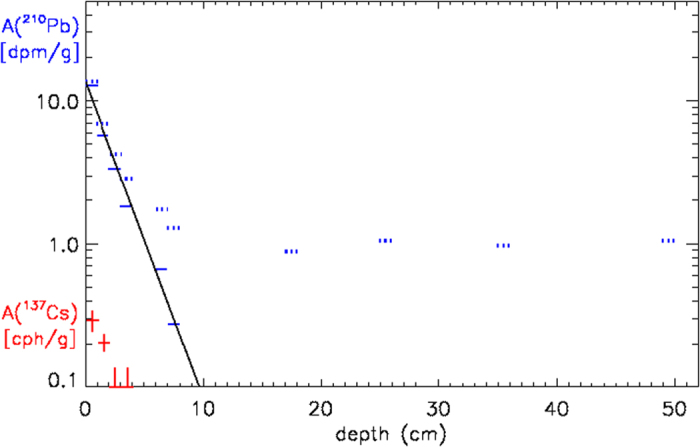
^210^Pb activity as a function of the sediment depth (data from Bonino *et al.*, 1993^21^). The sediment samples for the activity estimation have been taken at 10 different depths with a sampling thickness of 1 cm. Dashed and solid blue lines are the total and excess activity, respectively. The black line is the least-square fit of the excess activity. ^137^Cs activity is reported in the left-bottom corner.

The experimental measurements are shown in Fig. 5.

The decreasing solid line, which represents the least-square exponential fit of the excess activity, allowed to obtain the sedimentation rate S = 0.064 cm y^−1^ with an estimated error of 5% and correlation coefficient r^2^ = 0.98^21^. Moreover, in order to validate the results obtained by ^210^Pb method and to test the presence of the core top, Bonino *et al.* (1993) measured ^137^Cs activity (T_1/2_ = 30 yr) which is primarily due to nuclear bomb testing and related to the radioisotope concentration maximum in the atmosphere during the Sixties. The γ-activity measurement of ^137^Cs (see Fig. 5) was performed using a HPGe detector with relative efficiency of 25%, located at our underground Laboratory of Monte dei Cappuccini (INAF) in Torino. The activity maximum is revealed in the first sample, as expected on the basis of ^210^Pb dating. The result ensures that the core top is present and it has not been disturbed during drilling operations or by biological activity (at least within the sampling thickness).

### δ^18^O measurement

In order to obtain the δ^18^O values of the samples, taken with a spacing of 2.5 mm, we soaked 5 g of sediment in 5% calgon solution overnight, then treated it in 10% H_2_O_2_ to remove any residual organic material, and subsequently washed it with a distilled-water jet through a sieve with a 150 μm mesh. The fraction >150 μm was kept and oven-dried at 50 °C. The planktonic foraminifera Globigerinoides ruber were picked out of the samples under the microscope. For each sample, 20–30 specimens were selected from the fraction comprised between 150 μm and 300 μm. The use of a relatively large number of specimens for each sample removes the isotopic variability of the individual organisms, giving a more representative δ^18^O value. The stable isotope measurements were performed using a VG-PRISM mass spectrometer fitted with an automated ISO-CARB preparation device. Analytical precision based on internal standards was better than 0.1‰. Calibration of the mass spectrometer to VPDB scale was done using NBS19 and NBS18 carbonate standards.

### Temperature (T) and salinity (S) influence on δ^18^O

The theoretical δ^18^O_c_ in the foraminifera calcite was determined by using its relationship with temperature and δ^18^O_w_ of the water, given by Shackleton^15^, δ^*18*^*O*_*c*_ *=* *3.86 + *δ^*18*^*O*_*w*_ *− 0.23T* and the relationship derived by Pierre^16^ for Mediterranean surface waters: δ^*18*^*O*_*w*_ *= 0.25 S – 8.2*.

### δ^18^O-Po river discharge relationship

**Figure 6:**
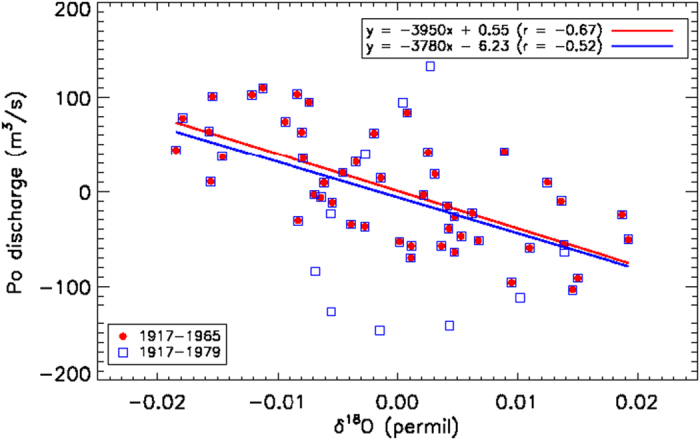
Linear regression between δ^18^O and Po River discharge decennial components over the period 1917–1979 (blue line) and 1917–1965 (red line).

The relationship between δ^18^O and Po discharge is based on data from 1917 to 1979 (top of our core). The Hydrological Office of the Po River-Parma started to regularly convert stage measurements into discharge estimates by using a discharge rating curve in 1917; discharge estimates available for the period 1807–1916 are not considered since they are based on stage measurements and on the 1917 discharge rating curve, hence subject to uncertainties related to river’s bed geometry^3^. The linear regression between these curves is represented by the blue line in Fig. 6. The value of the correlation coefficient (r = −0.52) indicates that for this sample of N = 49 points the correlation is significant above the 99% c.l. The regression does not change if we exclude from the fit the last part of the series, corresponding to the top of the core (see red line in Fig. 6). The extrapolation of Po discharges before 1917 is shown in main Fig. 3 for comparison with our reconstruction.

## Additional Information

**How to cite this article**: Taricco, C. *et al.* Marine Sediments Remotely Unveil Long-Term Climatic Variability Over Northern Italy. *Sci. Rep.* **5**, 12111; doi: 10.1038/srep12111 (2015).

## Supplementary Material

Supplementary Information
