# Supplementary material for: Marine Sediments Remotely Unveil Long-Term Climatic Variability Over Northern Italy
Source: Sci Rep. 2015 Jul 31;5:12111. doi: 10.1038/srep12111 (PMC4521202; doi:10.1038/srep12111)
Supplement: Supplementary Information [file srep12111-s1.doc]

**SUPPLEMENTARY INFORMATION for:**

**Marine Sediments Remotely Unveil Long-Term Climatic Variability Over Northern Italy**

Carla Taricco, Silvia Alessio, Sara Rubinetti, Davide Zanchettin, Simone Cosoli, Miroslav Gačić, Salvatore Mancuso, Angelo Rubino

1. **Spectral methods**

**1.1 Singular Spectrum Analysis (SSA).** The SSA methodology involves three basic steps: (a) embedding a time series of length *N* in a vector space of proper dimension *M*^33,34^; (b) computing the *M* x *M* lag-covariance matrix *C_D_* of the data (see the two different approaches of Broomhead and King^35^, and Vautard & Ghil^36^); and (c) diagonalizing *C_D_*: Λ_D_ = E_D_^T^C_D_E_D_, where Λ_D_ = *diag*(λ_1_,λ_2_,λ_3_,…λ_M_), with λ_1_>λ_2_>λ_3_>…>λ_M_>0, with and *E_D_* is the *M* x *M* matrix having the corresponding eigenvectors *E_k_*, *k*=1,*M* as its columns. For each *E_k_* we construct the time series, of length *N-M+1*, called the *k*-th principal component (PC); this PC represents the projection of the original time series on the eigenvector *E_k_* (also called empirical orthogonal function, EOF). Each eigenvalue *λ_k_* gives the variance of the corresponding PC; its square root is called singular value (SV). Given a subset of eigenvalues, it is possible to extract time series of length *N* by combining the corresponding PCs; these time series are called reconstructed components (RCs) and capture the variability associated with the eigenvalues of interest. In order to reliably identify the trend and oscillations in a series, the Monte Carlo method (MCSSA) is used^37^. In this approach, we assume a model for the analyzed time series (null-hypothesis) and we determine the parameters using a maximum-likelihood criterion. Then a Monte Carlo ensemble of surrogate time series is generated from the model and SSA is applied to data and surrogates (EOFs of the null-hypothesis basis are used), in order to test whether it is possible to distinguish the series from the ensemble. Since a large class of geophysical processes generate series with larger power at lower frequencies, we assume AR(1) noise in evaluating evidence for trend and oscillations. This is done to avoid overestimating the system predictability, by underestimating the amplitude of the stochastic component of the time series^37^. SSA is particularly useful for climatic time series^35^, which are most often short and noisy. The SSA methodology has in fact been applied to instrumental and proxy climate records; two review papers^34,38^ and references therein cover the methodology, as well as many applications.

**1.2 Continuous Wavelet Transform (CWT).** The Wavelet Transform (WT) allows an evolutionary spectral analysis of a series in the time-scale plane^39,40^. The concept of scale is typical of this method: the scale is a time duration that can be properly translated into a Fourier period and hence a frequency. The Continuous Wavelet Transform (CWT) in spectral applications is discretized by computing it at all available time steps and on a dense set of scales^41^. The square modulus of the transform expresses spectral density as a function of time and frequency (scalogram). A filtered version of the signal can then be reconstructed selecting only the contributions from a given set of periods. By time averaging the CWT at each value of period (scale), the Global Wavelet Spectrum (GWS) and the corresponding significance levels, using a background spectrum of red noise, can be computed, thus obtaining a time-averaged spectral estimate comparable with those obtained by classical methods. However, CWT is a multiresolution analysis: frequency resolution is high at low frequency and poor at high frequency^41^. It is therefore particularly suited for determining the frequency of oscillations in the low-frequency range of the spectrum and to reconstruct them accurately.

1. **Spectral analysis of δ^18^O series.**

The δ^18^O profile measured in carbonatic shells of foraminifera *Globigerinoides ruber*, taken from the shallow-water Ionian core GT90-3, consists of a continuous record of 560 points, from 200 BC to 1979 AD, with a sampling interval of Δt = 3.87 years (Fig. 1S).

**
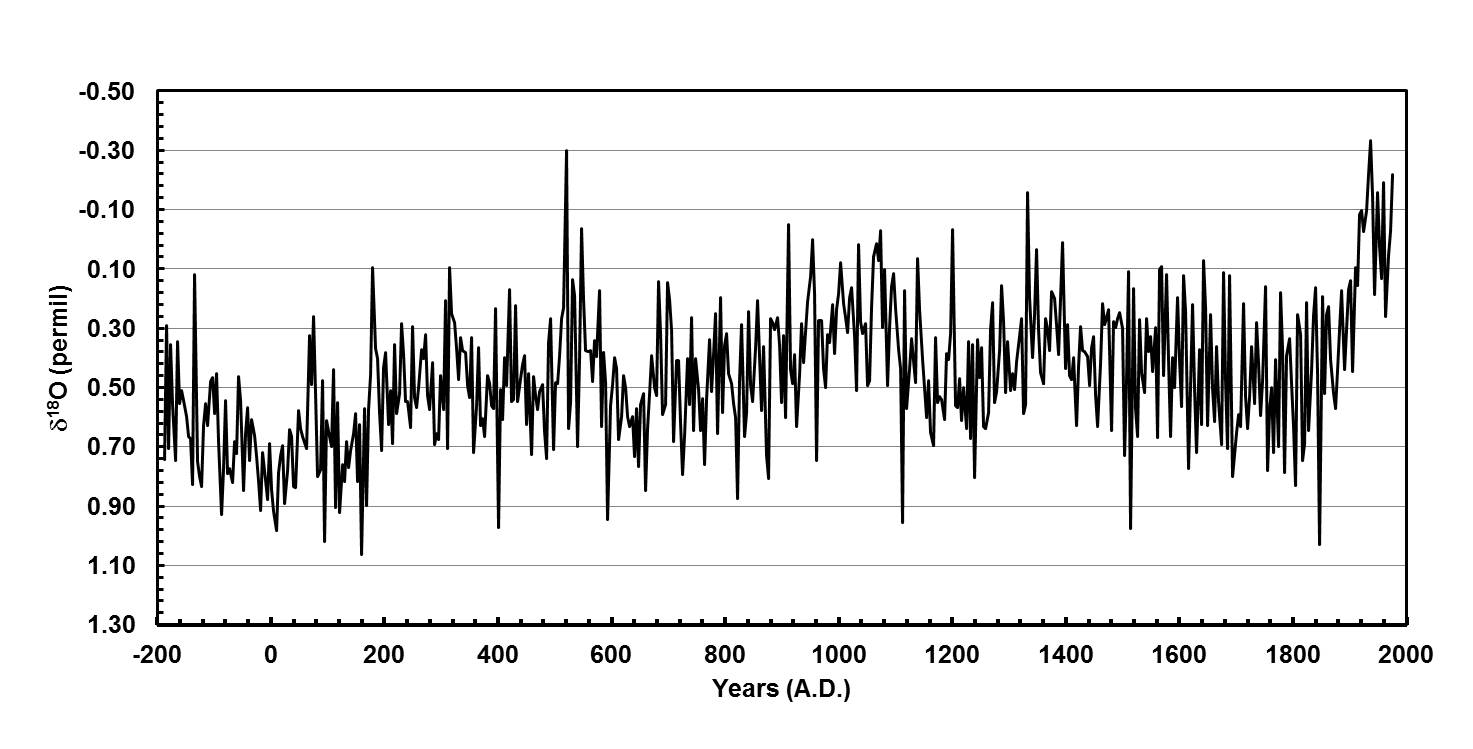
Fig. 1S δ^18^O profile measured in foraminifera Globigerinoides ruber, taken from the shallow-water Ionian core GT90-3.**

Taricco et al. (2009, ref. [15] of the main paper) discussed the features of this series and the results obtained by applying several advanced spectral methods to it. Since the focus here is on the decennial range of periods, we adopted a relatively short window (M = 50). In this case, the most powerful components are represented by the eigenvalues 1-6 (see the SSA spectrum in Fig. 2S), as confirmed by the Monte Carlo test, which is reported in the inset of the same figure. The periods associated to each significant component were determined by the Maximum Entropy Method (MEM). These periods (and corresponding variances) are equal to 770 years (26.7 %, RC 1), 300 years (9.3 %, RC 2), 180 years (4.5 %, RCs 3,8), 125 years ( 2.9 %, RC 4) and 11.4 years (4.6 %, RCs 5,6).


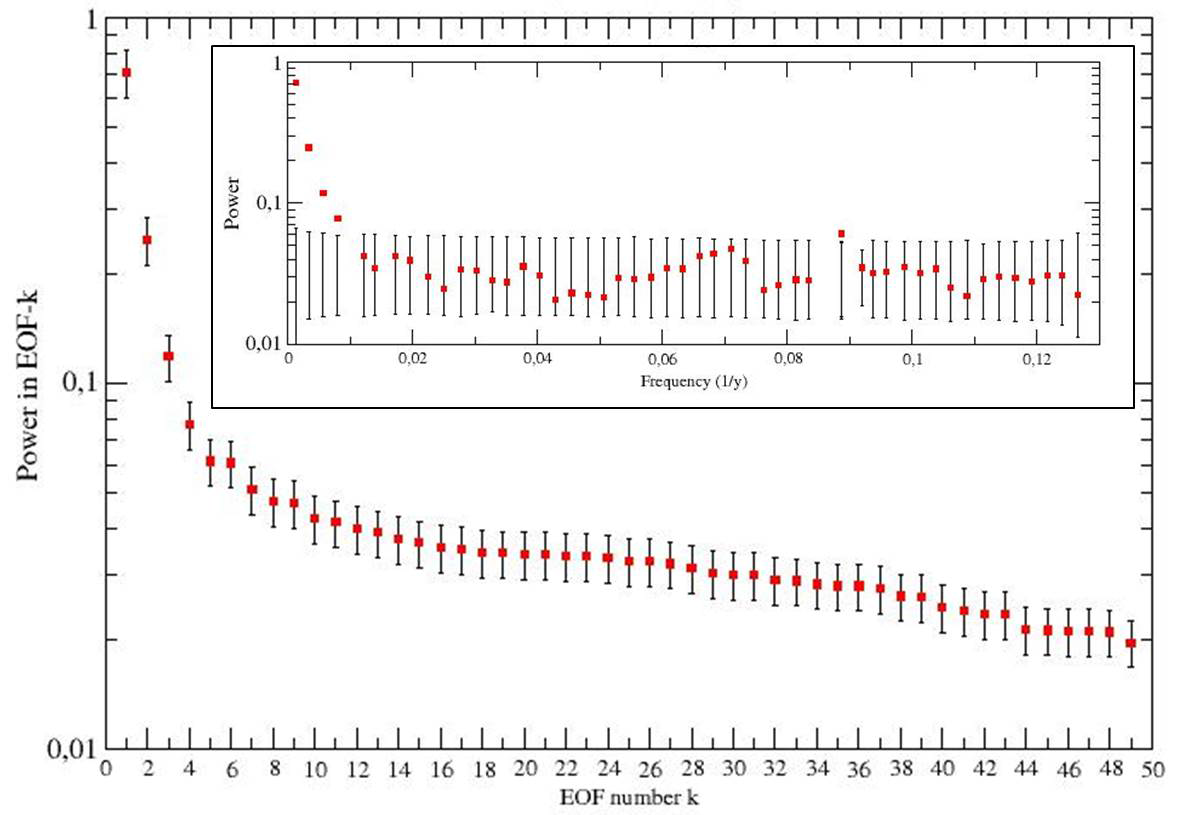


**Figure 2S: SSA power spectrum and Monte Carlo significant test of δ^18^O series.** Main panel: Eigenvalue spectrum of δ^18^O SSA obtained by a 50-point window. Inset: final step of Monte Carlo SSA test. The Monte Carlo ensemble size is 10,000. Null hypothesis includes an AR(1) model plus EOFs 1-6. No excursions occur outside the 99% limits, indicating that all the components are compatible with the null hypothesis.

The decennial component of the δ^18^O time series (see Fig. 3S) is a significant decadal variation at high confidence level (99%) and is present over the entire interval covered by the series. The average amplitude of this oscillation is 0.08 permil over 2,200 years.


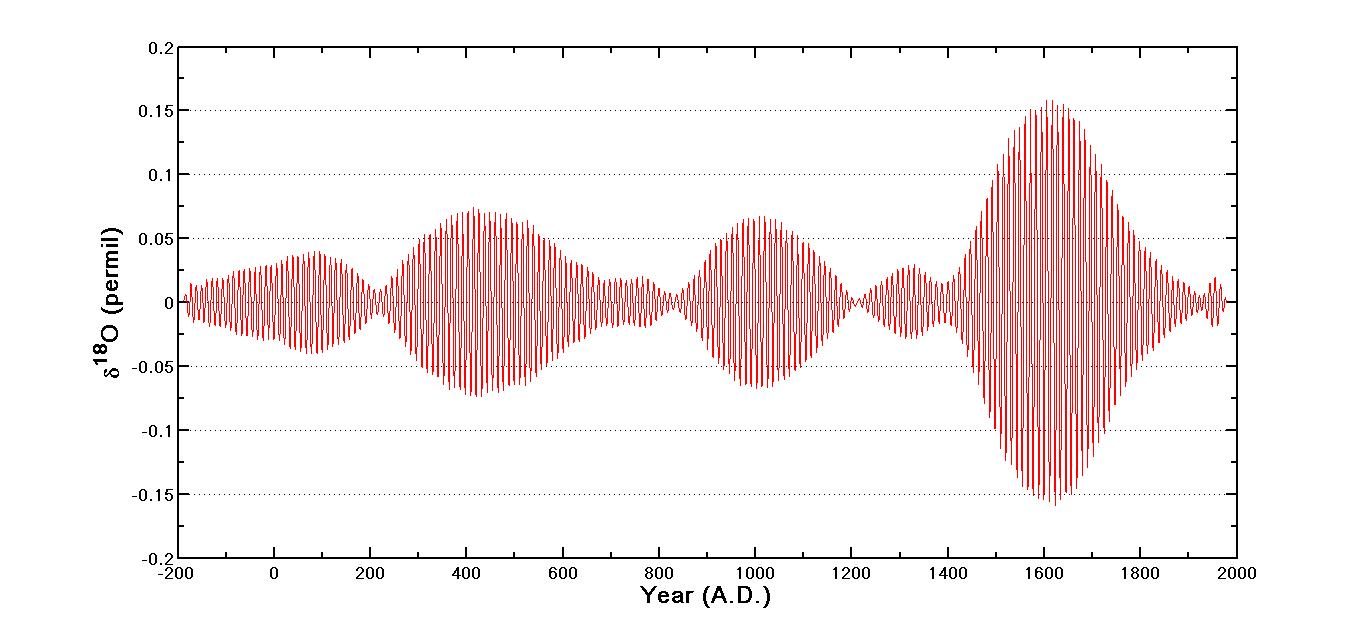
**Figure 3S:** δ^18^O **decadal component over the last 2200 years.**

1. **Spectral analysis of Po river discharge series.**

Spectral analysis is conducted of the 200-year Po annual discharge series (200 points; see Fig.2a). The time series thus includes the discharge estimates for the period 1807-1917, which are based on stage measures and on the stage-discharge rating curve of 1917 (see ref. [3] of main manuscript for details). We adopted a window width of *M* = 80 points, corresponding to a time window of 80 y. We obtained, however, coherent results for a fairly wide range of *M* values, from 40 to 100 points. The empirical orthogonal functions (EOFs) 1–5, 8 account for roughly 20% of the total variance in the time series. Monte Carlo-SSA allowed us to verify that the statistically significant part of the Po time series is given by the sum of these 6 components (which are significant at the 99% confidence level), with a residue of red noise. EOFs 1,2, EOFs 3,4 and EOFs 5,8 capture oscillatory components whose associated periods were determined by MEM. These periods (and corresponding variances) equal 12 y (7.5%), 3 y (6.3%) and 20 y (5.8%). In the Monte-Carlo test shown in Fig.3S, the error bars bracket 99% of the eigenvalues obtained by the SSA of 5000 surrogate series; these series are generated by a model that superposes EOFs 1–5, 8 onto a red-noise process. The eigenvalues that lie outside the error bars are only those associated with EOFs 1–5, 8, which have been included in the null hypothesis; this confirms that the model AR(1)+EOFs 1–5, 8 captures the variability of the Po time series at the 99% confidence level. We obtained this result after rejecting, at the same confidence level, a whole range of null hypotheses, including different combinations of EOFs.

The CWT analysis was performed using a complex Morlet wavelet with parameter *ω*_o_ = 6. The top panel of Fig. 4S shows the series, the bottom-left panel shows the scalogram as color-filled contour lines, with black contours indicating significant power at 95% c.l.. The white cup-shaped line is the cone of influence, outside which the wavelet power is affected by edge effects due to zero-padding of the series when the transform is computed in the frequency domain via FFT. The bottom-right panel shows the GWS and the related significance levels at 90, 95 and 98 % c.l.. CWT confirms the results previously obtained by Zanchettin et al. (refs [3],[5]) for the monthly Po series. It also indicates the same periodicities found by SSA (decennial and bi-decennial periodicities), but, due to the poor frequency resolution in the high frequency spectral range, it smooths too much the 3 year peak, that thus is only marginally significant. Moreover CWT reveals a 60 year oscillation; however, since this component is not revealed by both methods, we did not take it into account. In Fig. 2a, we show the decennial signal reconstructed by Inverse CWT (red curve), compared with that revealed by SSA (blue curve). The two reconstructions appear to be in good agreement, revealing the robustness of our analysis.


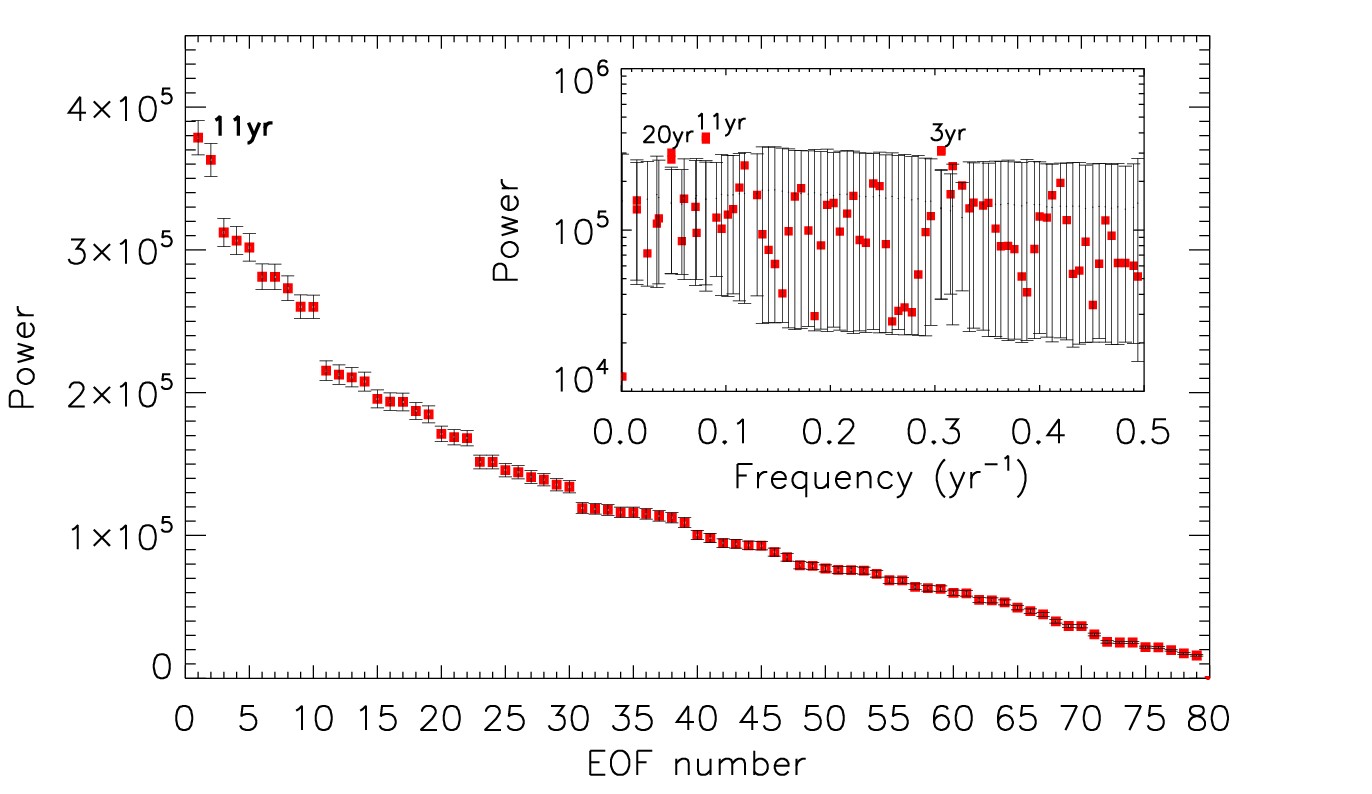


**Figure 3S: Main panel: Eigenvalue spectrum of SSA of the Po river discharge series obtained by a 80-point window.** Inset: final step of Monte Carlo SSA test. The Monte Carlo ensemble size is 10,000. Null hypothesis includes an AR(1) model plus EOFs 1-5,8.

**
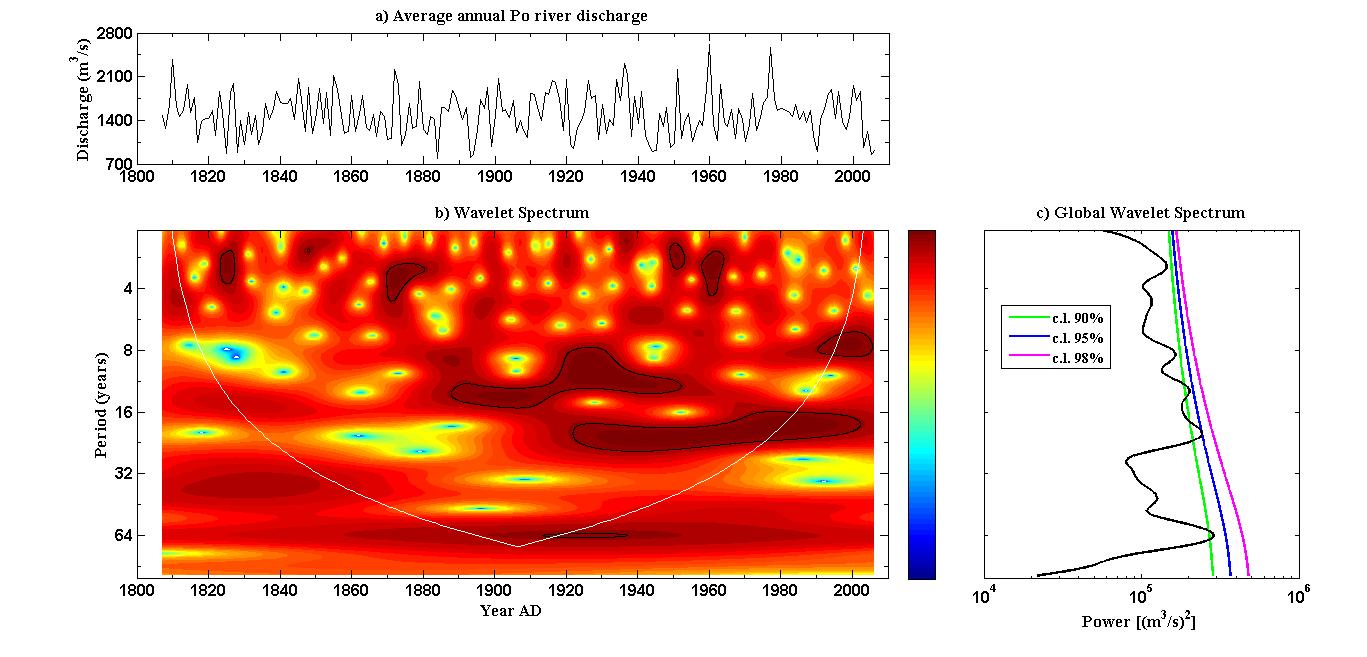
**

**Figure 4S: Wavelet analysis of the Po river discharge series.**

**4. Comparison between Po river discharge and surface salinity.**

In order to support the comparison shown in Fig. 2c, we calculated the correlation coefficient between Po discharges and seawater salinity in the layer 0-20 m of depth at different sites along the western Adriatic cost and at the Gallipoli site (see Figure 5S).


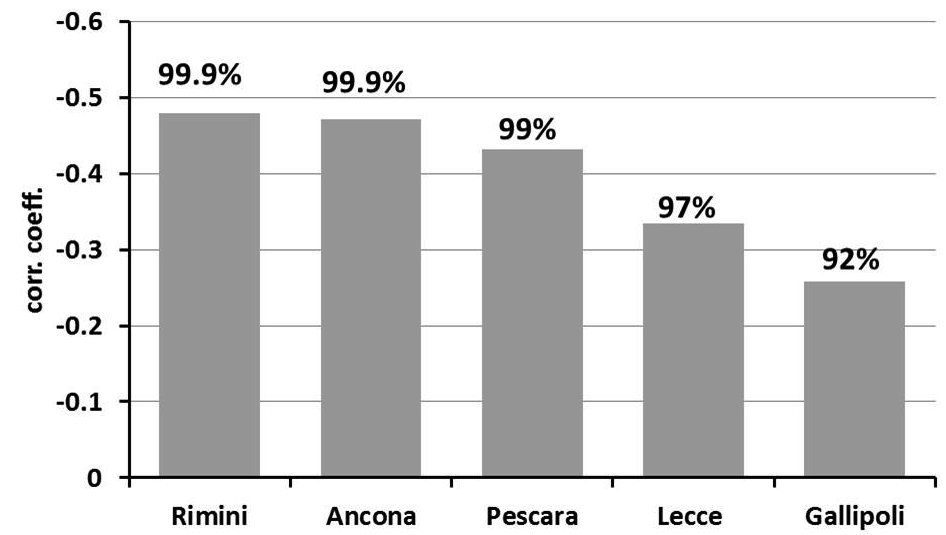


**Figure 5S: Correlation coefficient between Po and salinity at the layer 0-20 m at different sites. Numbers on top of the bars indicate the confidence level of the correlations.**

We notice that the correlation coefficient decreases, in its absolute value, southwards along the western Adriatic coast, starting from a value of about -0.5 at Rimini and Ancona (significant at 99% confidence).

The cross-correlation profiles in Figure 6S show that the absolute minimum of the correlation falls at lag zero. The consistent oscillatory behavior of the correlation profiles reflects the presence of decadal-scale variations in both the Po discharge series and all salinity series. It highlights the dominance of this component in all series during the considered period.


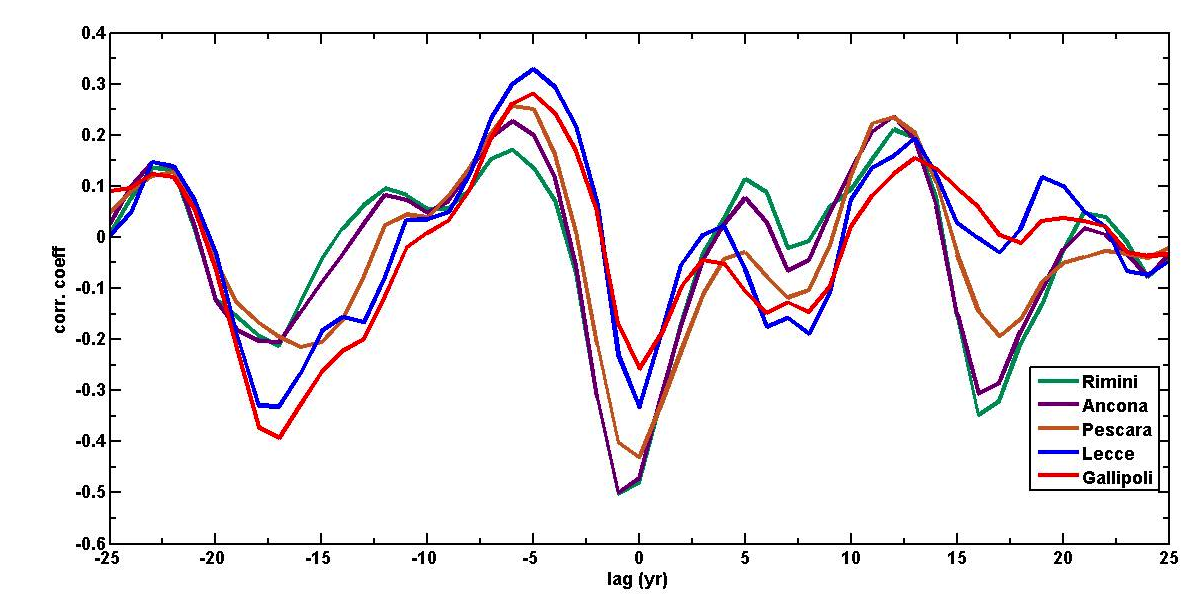


**Figure 6S: Cross-correlation between annual Po River discharge and annual near-surface salinity at different sites along the western Adriatic coast and at Gallipoli site**

**5. Comparison between Po River discharges and discharges from other Alpine rivers.**

The Po River, with an average discharge of about 1500 m^3^/s (see ref [3] of the main paper), represents the major source of freshwaters to the Adriatic and contributes to about half of the total inflow (e.g., ref [4] of the main paper). Other major Alpine rivers, such as the Adige, whose average discharge at Boara Pisani for the period 1922-1986 is 226 m^3^/s, the Brenta, whose average discharge at Barzizza for the period 1955-2001 is 66 m^3^/s, and the Piave, whose average discharge at Nervesa for the period 1955-2001 is 73 m^3^/s, closely follow the variability of the Po: Annual-average discharges of these rivers significantly correlate with the annual-average discharge of the Po (p-value of the correlation, r, is provided in brackets):

r_Po-Adige_ = 0.71873 (1.4537e-010)

r_Po-Brenta_ = 0.72667 (7.4868e-008)

r_Po-Piave_= 0.60488 (2.2053e-005)

Other Alpine rivers for which annual-average discharge data could not be retrieved, include Isonzo (average discharge at the mouth of about 170 m^3^/s), Livenza (average discharge at the mouth of about 85 m^3^/s) and Tagliamento (average discharge at the mouth of about 70 m^3^/s).

Hence, the freshwater inflow from the Po River represents itself one of the known major drivers of surface circulation in the Adriatic Sea (about the dominant character of the Po on the Adriatic^42^), but it is also representative of the interannual and lower-frequency variability of the total freshwater inflow from Alpine rivers, which in turn dominate the interannual and lower-frequency variability of the total riverine inflow into the Adriatic Sea.

**6. Correlation of Po River discharges and salinity anomalies in the Gulf of Taranto with the large-scale circulation and with surface freshwater fluxes**

Po River discharges are known to be affected by large-scale circulation anomalies, see, e.g., the connection with the North Atlantic Oscillation discussed in refs. [3,4] of the main paper. The connection is especially strong in winter and in the shouldering seasons (when peaks in the annual Po regime occur). The anomalous atmospheric pattern entails a continental-scale cyclonic anomaly spreading over the eastern North Atlantic, Europe and the Mediterranean (Figure 7S). Over the Ionian and Adriatic seas, the anomaly corresponds to southerly Sirocco-like conditions.

Concerning the net freshwater surface fluxes (Figure 8S), there is no robust correlation over the northern Ionian Sea and in the Gulf of Taranto linked to Po River discharges. There is, however, a significant signal near the Gulf of Taranto during spring and, more locally, also in summer. These results indicate that Po River discharges and freshwater surface fluxes in the Gulf of Taranto do not co-vary tightly: at best, during spring and summer, the net freshwater flux in the gulf of Taranto shares only less than 20% of variability with the Po River discharge.


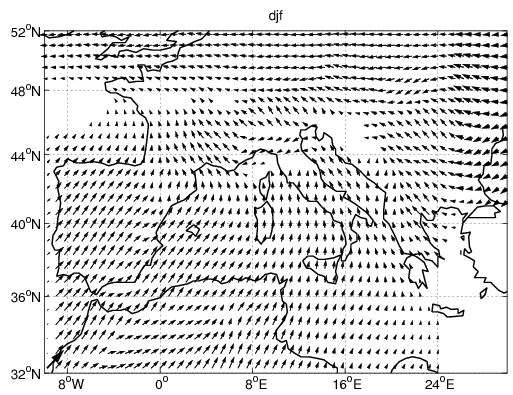

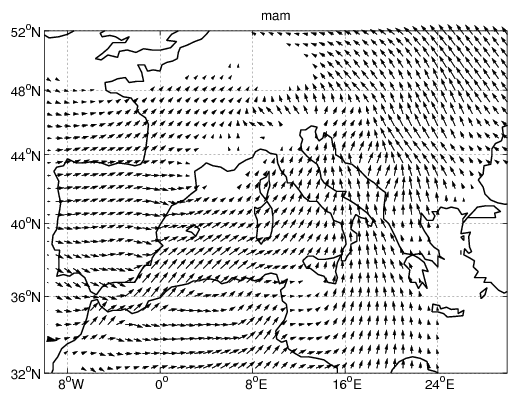

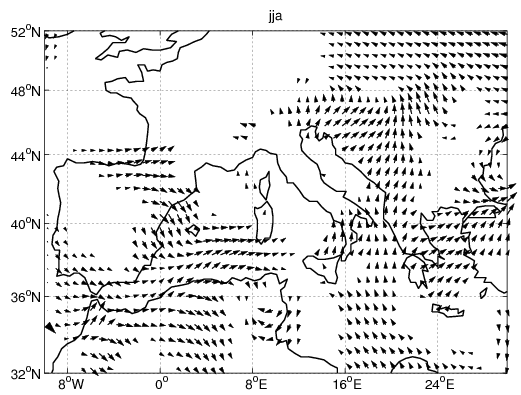

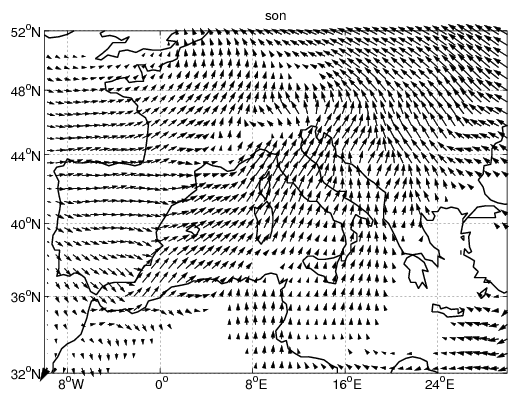


**Figure 7S - Correlation between seasonal Po River discharge and seasonal zonal and meridional 10 m wind data from ERA-Interim^43^ (period 1979-2014).** Arrows are shown for grid points where the correlation is statistically significant (p<0.05 accounting for autocorrelation) for at least one of the wind components. The direction and length of the arrows are determined by the strength of the correlations. Data are linearly detrended before analysis.


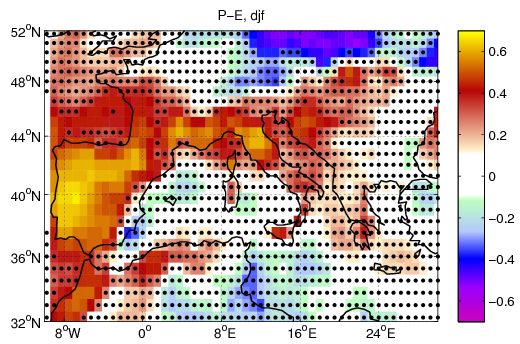

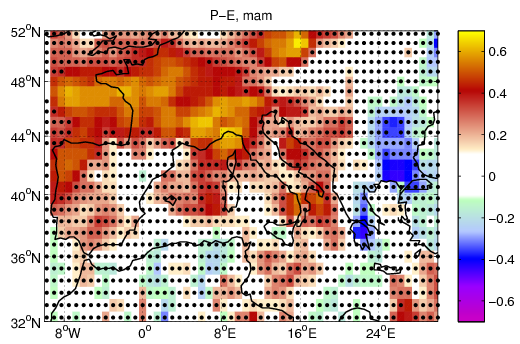

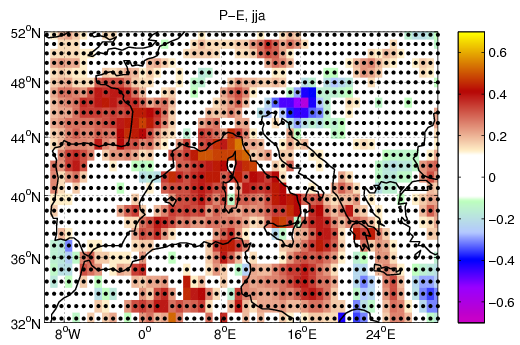

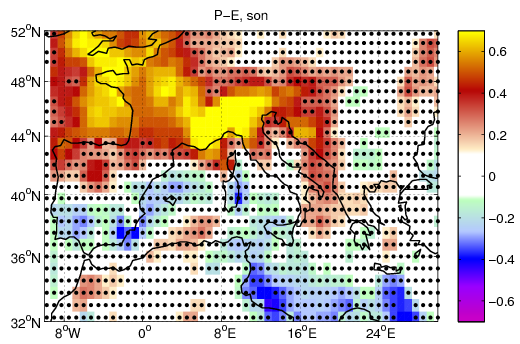


**Figure 8S - Correlation between seasonal Po River discharge and seasonal net surface freshwater fluxes (precipitation minus evaporation) from ERA-Interim^43^ (period 1979-2014)** Black dots mark grid points where the correlation is statistically not significant (p>0.05) accounting for autocorrelation in the data. Data are linearly detrended before analysis.


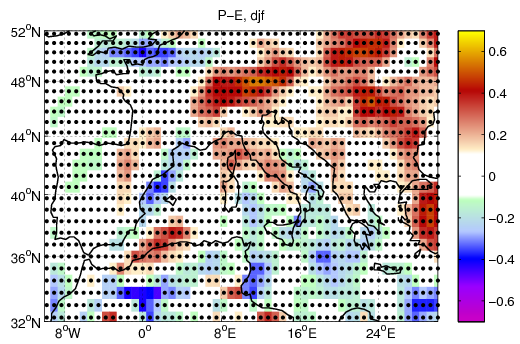

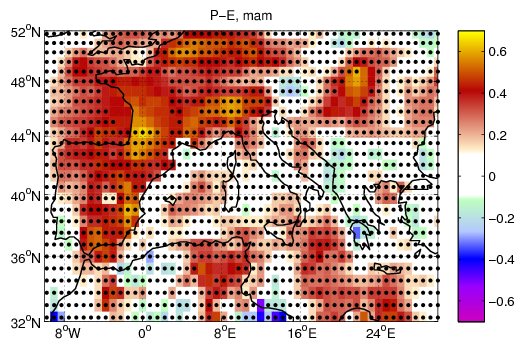


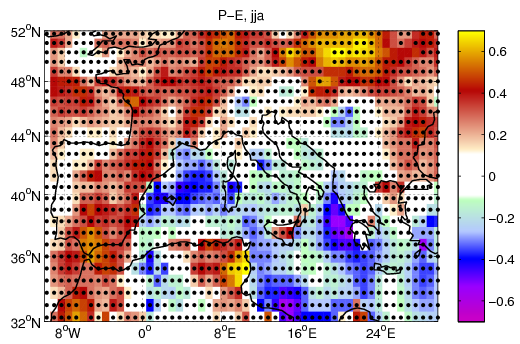

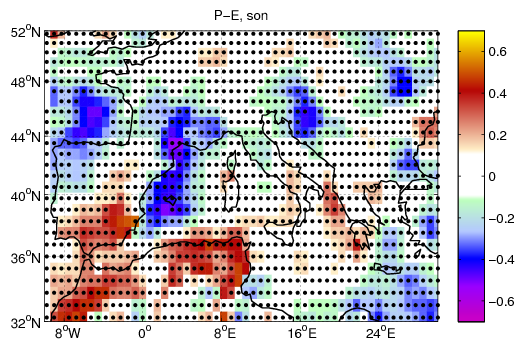


**Figure 9S - Correlation between annual surface salinity in the Gulf of Taranto (near Gallipoli) and seasonal net surface freshwater fluxes (precipitation minus evaporation) from ERA-Interim^43^ (period 1979-2002).** Black dots mark grid points where the correlation is statistically not significant (p>0.05) accounting for autocorrelation in the data.

Correlation between surface freshwater fluxes with the surface salinity observed near Gallipoli (Figure 9S) allows assessing whether the two variables significantly correlate, i.e., if local evaporation and precipitation are major factors contributing to upper-ocean salinity variations in the Gulf of Taranto. Figure 9S suggests that changes in the surface salinity in the Gulf of Taranto are not likely to be dominated by local surface freshwater fluxes. There are clear seasonal changes in the strength as well as in the sign of the correlations. Using yearly averages of freshwater fluxes results in non-significant, very small correlations over the Gulf of Taranto region. There are significant correlations off the western coast of Greece for the net freshwater flux during summer, but we similarly observe significant precipitation and precipitation minus evaporation signals over northern Italy during spring.

Merging information from the different results illustrated above and the tight correlation observed between Po River discharge and surface seawater salinity in the Gulf of Taranto discussed in the main paper, we summarize that:

- Variability of Po River discharges is significantly correlated with seawater salinity in the Gulf of Taranto;
- Variability of Po River discharges is linked to large-scale circulation anomalies, but the latter do not relate with significant freshwater surface fluxes over the Gulf of Taranto;
- Variability observed in surface seawater salinity in the Gulf of Taranto is not significantly correlated with variability in local freshwater surface fluxes.


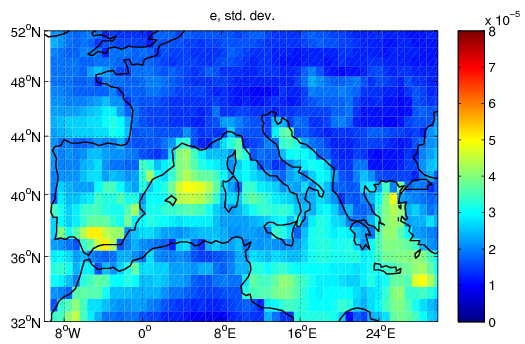

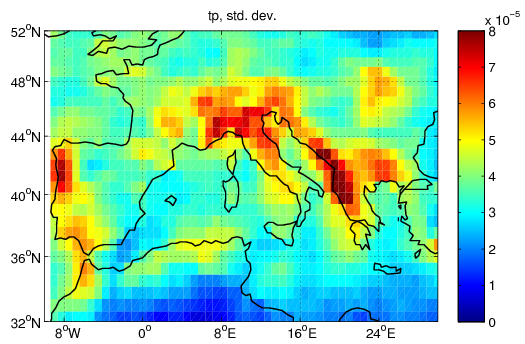

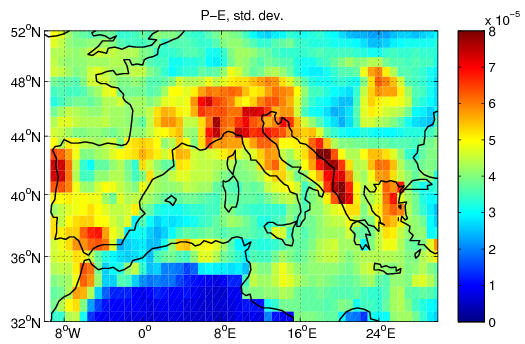


**Figure 10S – Standard deviations of annual-average freshwater fluxes at the surface from ERA-Interim.** Data are in meters of water equivalents and based on monthly-mean diagnostics for the period 1979-2014.

Furthermore, the amplitude of variations observed in evaporation, precipitation and net freshwater fluxes indicate that precipitation and evaporation contribute similarly to the freshwater flux variability in the northern Ionian Sea, with a slightly larger contribution from the former variable (Figure 10S). This result agrees with indications by Romanou et al. (2010)^44^ that “evaporation over the Ionian waters is nearly uniform, with values ranging from 3 mm day in the northern part of the basin near the Straits of Otranto to 3.5 mm day near the Libyan coast. Precipitation, however, exhibits a preferentially zonal structure with the largest values occurring in the northern Ionian Sea (1.4 mm day) south of the Straits of Otranto, although the interannual variability of this signal is also the largest in the basin (about 2.3 mm day; Fig. 2f). The freshwater budget in the Ionian basin is mainly controlled by the rainfall patterns in the region rather than the evaporation, which is mostly uniform.”

**Supplementary references:**

1. Vautard, R., Yiou, P. & Ghil, M. Singular-spectrum analysis: A toolkit for short, noisy chaotic signals. *Physica D* **58,** 95–126 (1992).
2. Ghil, M. *et al*. Advanced spectral methods for climatic time series. *Rev. Geophys*. **40,** 3.1–3.41 (2002).
3. Broomhead, D.S. & King, G.P. Extracting qualitative dynamics from experimental data. *Physica D* **20,** 217–236 (1986).
4. Vautard R. & Ghil M. Singular spectrum analysis in nonlinear dynamics with applications to paleoclimatic time series. *Physica D* **35,** 395–424 (1989).
5. Allen, M.R. & Smith, L.A. Monte Carlo SSA: detecting irregular oscillations in the presence of coloured noise. *J. Clim.* **9,** 3373-3404 (1996).
6. Ghil, M. & Taricco, C. Advanced spectral analysis methods. *Past and Present Variability of the Solar-terrestrial System: Measurement, Data Analysis and Theoretical Models* edited by: Cini Castagnoli, G. and Provenzale, A., IOS Press, Amsterdam, The Netherlands, 137–159 (1997).
7. Kumar, P. & Foufoula-Georgiou, E. Wavelet Analysis for Geophysical Applications. *Rev.* *Geophys.* **35,** 385–412 (1997).
8. Percival, D.B. & Walden, A.T. Wavelet Methods for Time Series Analysis. *Cambridge University Press* (2000).
9. Torrence, C. & Compo, G.P. A practical guide to wavelet analysis. *Bull. Amer. Meteor. Soc*. **79,** 61–78 (1998).
10. Cushman-Roisin, B., Malačič, V. & Gačić, M. Tides, seiches and low-frequency oscillations. In: Physical Oceanography of the Adriatic Sea. B. *Cushman-Roisin et al*., eds., Kluwer Academic Publ., 217-240, (2001).
11. Dee, D. P., and 35 co-authors: The ERA-Interim reanalysis: Configuration and performance of the data assimilation system. *Quart. J. R. Meteorol. Soc*., **137**, 553-597. DOI: 10.1002/qj.828 (2011).
12. Romanou, A., Tselioudis, G., Zerefos, C.S., Clayson, C-A., Curry, J.A. & Andersson, A. Evaporation–Precipitation Variability over the Mediterranean and the Black Seas from Satellite and Reanalysis Estimates. *J. Climate*, **23**, 5268–5287, doi: <http://dx.doi.org/10.1175/2010JCLI3525.1> (2010).
